# Hypertensive Crisis, Burden, Management, and Outcome at a Tertiary Care Center in Karachi

**DOI:** 10.1155/2014/413071

**Published:** 2014-08-18

**Authors:** Aysha Almas, Ayaz Ghouse, Ahmed Raza Iftikhar, Munawwar Khursheed

**Affiliations:** ^1^Department of Medicine, Stadium Road, Aga Khan University, P.O. Box 3500, Karachi 74800, Pakistan; ^2^Aga Khan University Medical College (AKUMC), Karachi 74800, Pakistan; ^3^Emergency Medicine, Aga Khan University, Karachi 74800, Pakistan

## Abstract

*Objectives*. Hypertension, if uncontrolled, can lead to hypertensive crisis. We aim to determine the prevalence of hypertensive crisis, its management, and outcome in patients presenting to a tertiary care center in Karachi. *Methods*. This was a cross-sectional study conducted at the Aga Khan University, Karachi, Pakistan. Adult inpatients (>18 yrs) presenting to the ER who were known hypertensive and had uncontrolled hypertension were included. *Results*. Out of 1336 patients, 28.6% (387) had uncontrolled hypertension. The prevalence of hypertensive crisis among uncontrolled hypertensive was 56.3% (218). Per oral calcium channel blocker; 35.4% (137) and intravenous nitrate; 22.7% (88) were the most commonly administered medication in the ER. The mean (SD) drop in SBP in patients with hypertensive crisis on intravenous treatment was 53.1 (29) mm Hg and on per oral treatment was 43 (27) mm Hg. The maximum mean (SD) drop in blood pressure was seen by intravenous sodium nitroprusside; 80 (51) mm Hg in SBP. Acute renal failure was the most common complication with a prevalence of 11.5% (24). *Conclusion*. The prevalence of hypertensive crisis is high. Per oral calcium channel blocker and intravenous nitrate are the most commonly administered medications in our setup.

## 1. Background

Hypertension is a common chronic medical condition affecting people in Pakistan and the rest of the world [1]. It is an important modifiable risk factor for cardiovascular morbidity and mortality, particularly for stroke (accounting for 51% of all stroke deaths worldwide), ischemic heart disease (45% of all deaths), chronic kidney disease, congestive heart failure, aortic aneurysm, and peripheral arterial disease [2]. Prevalence of hypertension (systolic blood pressure >140 mm Hg or diastolic blood pressure >90 mm Hg, or on antihypertensive medications) in Pakistan has increased from 17% in 1980 to 35% in 2008 in adults aged 18 years and older [3]. The increasing prevalence of hypertension together with a deficient control makes this one of the frequent conditions that require urgent medical attention [4].

The prevalence of uncontrolled hypertension varies around the world, with the lowest prevalence in rural India (3.4% in men and 6.8% in women) and the highest prevalence in Poland (68.9% in men and 72.5% in women) [5]. Recent population based data for control rates of hypertension from Pakistan are not available. However, the control of hypertension was 23% from a community based data in urban population from Karachi, Pakistan [6]. Uncontrolled hypertension can progress to hypertensive crisis defined as a systolic blood pressure >180 mm Hg or a diastolic blood pressure >120 mm Hg [7]. Hypertensive crisis can be further classified as a hypertensive urgency or hypertensive emergency depending on end-organ involvement including cardiac, renal, and neurologic injury. “*Hypertensive urgency*” refers to severe hypertension without evidence of new or worsening end-organ injury while “*Hypertensive emergency*” refers to a severe hypertension that is associated with new or progressive end-organ damage [8].

Hypertensive crises (76% urgencies, 24% emergencies) represented more than one-fourth of all medical urgencies/emergencies. Hypertensive urgencies frequently present with headache (22%), epistaxis (17%), and psychomotor agitation (10%) and hypertensive emergencies frequently present with chest pain (27%), dyspnea (22%), and neurological deficit (21%) [9]. The reason for uncontrolled hypertension in Pakistan is high due to lack of awareness, knowledge, adherence, and attitudes of Pakistani patients with hypertension [10]. However there is no data on patients with hypertensive crisis from tertiary care hospitals in Pakistan. Additionally the number of patients who complicate towards stroke, myocardial infarction, and chronic kidney disease is also not known. Hence, it is essential to have figures on prevalence and clinical presentation from our setup. Therefore, we conducted this study to determine the prevalence of hypertensive crisis, its management, and its outcome in patients presenting to a tertiary care center in Karachi.

## 2. Methods

### 2.1. Study Design and Study Population

This was a retrospective study conducted at the Aga Khan University, Karachi, Pakistan. The Aga Khan University Hospital (AKUH) has 563 beds in operation and provides services to over 50,000 hospitalized patients and to over 600,000 outpatients annually. Ethical approval from the ethics review committee of the Aga Khan University (1985-Med-ERC-11) was taken for conduct of the study.

Adult inpatients (>18 yrs) presenting to the ER who were known hypertensive and had uncontrolled hypertension were included. Controlled blood pressure was defined as systolic blood pressure (SBP) <140 mm Hg or diastolic blood pressure (DBP) <90 mm Hg [11]. Uncontrolled hypertension was defined as SBP >140 mm Hg and DBP >90 mm Hg in both diabetic and nondiabetic patients who were either aware of their problem or under pharmacological treatment [12]. The sample was drawn using computerized medical record system International Classification of Diseases-9-Coordination and Maintenance (ICD-9-CM) at health information management system in the hospital. Patients admitted with primary diagnosis of hypertension crisis, uncontrolled hypertension, hypertensive emergency, and hypertensive urgency were selected through the ICD-9-CM (I-10: essential (primary) hypertension, I-11: hypertensive heart disease, I-12: hypertensive renal disease, I-13: hypertensive heart and renal disease, and I-15: secondary hypertension). Patients whose medical records did not contain minimal clinical information to allow case classification (hypertensive urgency or emergency) were excluded from the study [13]. Data over a period of 5 years, from year 2005 till year 2010, was used. A sample of 1336 consecutive patients fulfilling the inclusion criterion was selected. All patients gave a general consent on admission; however informed consent was not taken as data was extracted later through ICD-9-CM.

### 2.2. Study Variables and Measurements

Data on demographics, comorbid conditions, clinical symptoms, blood pressure readings at subsequent time intervals, length of stay, and antihypertensive drug therapy was recorded by trained data collectors. Age and gender were recorded as mentioned at the time of admission. A history of physician-diagnosed diabetes mellitus (DM), chronic kidney disease (CKD), ischemic heart disease (IHD), and stroke was noted from the patient's medical record file. DM was defined as fasting plasma glucose ≥126 mg/dL at a prior visit [14]. CKD was defined as rise in serum creatinine of >1.2 mg/dL for 3 months [15]. IHD was diagnosed using WHO definition [16]. Clinical symptoms were recorded from the physician's initial assessment sheet.

Blood pressure readings, at different time intervals, were recorded from vital sheets for nursing services. Blood pressure was measured using a mercury sphygmomanometer with the patient in sitting position. Hypertensive crisis was defined as a systolic blood pressure >180 mm Hg* or* a diastolic blood pressure >120 mm Hg [7]. Management of patient was assessed by recording the list of medication from the computer generated pharmacy sheet attached inside the medical record file. Antihypertensive treatment was divided into two types: medication given per oral and medications given intravenously. Troponin-I values where available were recorded using the Patient Profile Viewer, an online hospital database software.

Outcome of patient was assessed in terms of length of stay and complications. Mean length of stay was recorded by calculating the time interval between the patient's admission date/time and discharge date/time from ER/ward. Various complications like myocardial infarction, stroke, aortic dissection, acute renal failure, and pulmonary edema developed during the hospital stay were recorded using the discharge summary notes filled by the primary consultant. Myocardial infarction was diagnosed when blood levels of sensitive and specific biomarkers such as cardiac troponin or CKMB are increased in the clinical setting of acute myocardial ischemia with electrocardiographic changes [17]. According to WHO definition, stroke was defined as a rapidly developing clinical sign of focal (at times global) disturbance of cerebral function, lasting more than 24 hours or leading to death with no apparent cause other than that of vascular origin [18]. Aortic dissection was referred as the condition when a separation has occurred in aortic wall intima as diagnosed on CT scan, causing blood flow into a new false channel composed of the inner and outer layers of the media [19]. Acute renal failure was diagnosed when the plasma urea nitrogen (PUN) or serum creatinine did not stabilize within 72 hours [20]. Acute pulmonary edema was defined as alveolar or interstitial edema verified by chest X-ray and/or with O_2_ saturation <90% on room air prior to treatment accompanied by severe respiratory distress, with crackles over the lungs and orthopnea [21]. A minimum sample size of 237 patients was required to estimate a proportion of 19% of patients with hypertensive crises presenting to ER, with bound on error of 5% and alpha of 5%.

### 2.3. Statistical Methods

Data was analyzed using Statistical Package of Social Sciences (SPSS) version 19.1. Mean and standard deviation were used for qualitative variables and frequency and percentage for qualitative variables. Chi-square test was used to compare categorical variables and Student's* t*-test was used to compare quantitative variables.

## 3. Results

### 3.1. Baseline Characteristics of Patients

A total of 73,063 hypertensive patients presented to the ER between years 2005 and 2010 out of which a sample of 1336 (1.8%) patients was taken for this study. The prevalence (%) of uncontrolled hypertension was 28.9% (387). Prevalence of hypertensive crisis overall was 16.3% (218) and among those with uncontrolled hypertension is 56.3% (218). Mean age (SD) of patients presenting to the ER was 56.7 (14.7) and 175% (45.2) of patients were male. Overall, dyslipidemia was the most common comorbidity in patients presenting with uncontrolled hypertension to the ER with the prevalence of 43.2% (167) followed by diabetes mellitus, 36.9% (143), and ischemic heart disease, 21.4% (83), and 13.9% (54) of them were smokers. The baseline characteristics of patients overall, with hypertensive crisis and without hypertensive crisis, are given in Table 1.

### 3.2. Clinical Characteristics of Patients

Headache was the most common presenting symptom, 35.7% (138), followed by dyspnea, 32.6% (126), chest pain, 21.4% (83), dizziness, 21.2% (82), vomiting, 17.3% (67), epistaxis, 5.2% (20), and neurologic deficit, 3.6% (14). On comparison of patients with hypertensive crisis with those with no hypertensive crisis the clinical symptoms that were statistically significant were as follows: headache was present in 42.2% (92) versus 27.2% (46) *P* value = 0.002 and chest pain was present in 17.4% (38) versus 26.6% (45) *P* value = 0.02.

**Figure 1:**
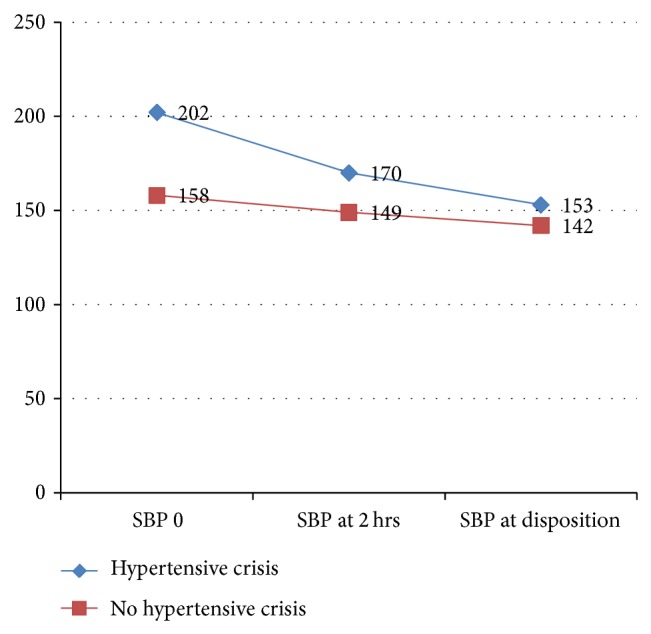
Trend of systolic blood pressure in ER.

**Figure 2:**
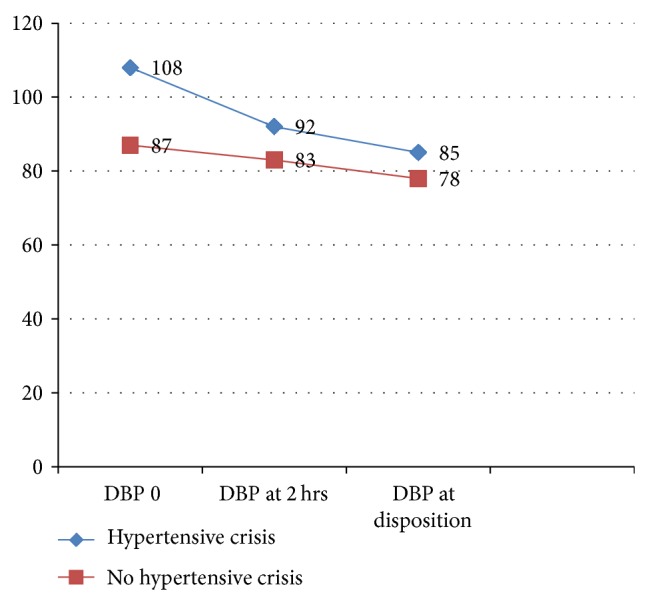
Trend of diastolic blood pressure in ER.

*Blood Pressure Trends of Patients with Hypertensive Crisis and without Hypertensive Crisis.* The mean (SD) systolic blood pressure (SBP) recorded in patients with hypertensive crisis versus no hypertensive crisis in ER was 202 (17.971) and 158 (13.387) (*P* value ≤ 0.001). The mean (SD) diastolic blood pressure in patients with hypertensive crisis versus no hypertensive crisis in ER was 108 (17.429) mm Hg and 87 (14.984) mm Hg, respectively, (*P* value ≤ 0.001). The trend of blood pressure recorded in the ER is shown in Figures 1 and 2.

### 3.3. Management of Patients with Hypertensive Crisis

*Antihypertensive Medications Used in Management of Patients with Hypertensive Crisis*. Calcium channel blocker was the most widely used oral antihypertensive medication in the ER, 35.4% (137). Intravenous (IV) nitrate was the most commonly administered IV medication in ER, 22.7% (88). The mean (SD) drop in SBP in patients with hypertensive crisis who received intravenous medications versus oral medications was 53.1 (29) mm hg and 43 (27), respectively (*P* value = 0.01). The mean drop in DBP in patients with hypertensive crisis who received intravenous versus perioral medications was 25.8 (19) and mm hg and 17.8 (22) mm Hg, respectively (*P* value = 0.006). Comparison of blood pressure trends in patients with and without hypertensive crisis on intravenous or per oral medication is shown in Table 2. The maximum drop in systolic blood pressure and diastolic blood pressure was achieved by sodium nitroprusside: 80 (15) mm Hg and 37.5 (7.77) mm Hg. Types of intravenous medications and drop in SBP and DBP are shown in Table 3.

### 3.4. Outcome of Patients with Hypertensive Crisis

#### 3.4.1. Length of Stay

The total length of stay (in days) for patients with hypertensive crisis was 2.46 (0.164) whereas the total length of stay (in days) for patients without hypertensive crisis was 2.20 (0.158).

#### 3.4.2. Complications

Overall prevalence (%) of complications was 47.7% (104) in patients with hypertensive crisis. Acute renal failure was the most common complication with the prevalence (%) of 41.3% (43) followed by myocardial infarction 28.8% (30) and pulmonary edema 18.26% (19). Stroke accounted for 6.5% (12) of complications. On comparison of patients with hypertensive crisis and patients without it, myocardial infarction occurred in 7.2% (15) versus 9% (15), stroke occurred in 2.4% (5) versus 4.2% (7), and acute kidney injury occurred in 11.5% (24) versus 11.4% (19) patients, respectively.

On further categorization of patients with hypertensive crisis who received intravenous antihypertensive in ER versus not receiving intravenous medication, there was no significant difference in the complications in both groups.

## 4. Discussion

We have shown in this study on patients presenting to ER with hypertensive crisis that the prevalence of hypertensive crisis is high (56.3%). Headache was the most common symptom in patients presenting with this condition. The most potent intravenous medication for dropping blood pressure was sodium nitroprusside. Mean length of stay was longer in patients with hypertensive crisis and acute renal failure was the most common complication in these patients.

Our study showed a prevalence of hypertensive crisis of 56%. The incidence of hypertensive crisis was 47.22% in a study conducted in Bosnia and 16% in a study conducted in Brazil [22, 23]. This indicates that we see more hypertensive crisis compared to the above studies. The reasons for high prevalence of patients with hypertensive crisis in our setup are multiple. Lack of knowledge about control of hypertension and poor compliance to antihypertensive medications is a major issue in Pakistan [10]. Also, lack of proper health infrastructure in public sector leading to inability of the poor population to access healthcare adds onto the severity of hypertension in these patients [24].

In previous studies [25, 26], the most frequent clinical sign of patients presenting to the ER was headache (22% and 42%) and dizziness was reported among 30% of emergency department patients [25]. This study showed (36%) headache as the most common clinical sign followed by dizziness (21%) which supports the previous findings about the characteristics of hypertensive crisis patients. Complications of hypertensive crisis reported in previous studies conducted in Bahrain and Italy were acute coronary syndrome (32%), left ventricular heart failure (38%), and stroke (29.3%) [27] and cerebral infarction (24%), pulmonary edema (23%), and hypertensive encephalopathy (16%) [26]. However acute renal failure, myocardial infarction, and pulmonary edema are the most common complications in our setup. The reason for high prevalence of acute renal failure in our setup may be the higher number of patients with hypertensive emergency.

The most common intravenous medication used to control hypertensive crisis in this study was nitrates. Recommendation for use of intravenous nicardipine was reported by a study conducted in 2005 [28] whereas sodium nitroprusside, because of its direct vasodilator effect and immediate onset of action, was recommended for hypertensive emergency in a subsequent study in 2006 [29]. We observed in this study that sodium nitroprusside was the most potent intravenous antihypertensive in dropping blood pressure. However it was not the most commonly used drug in this study. In a similar study, intravenous labetalol was reported as the most frequently used antihypertensive medication for emergency department patients presenting with hypertensive urgency [25]. This indicates that the use of intravenous nitrate as the most common antihypertensive for management of hypertensive crisis (apart from use in patients with cardiac cause) is not completely in accordance with the recommendations. The most common oral antihypertensive used in our setup is calcium channel blocker. However in some studies, oral labetalol [25], beta blockers, diuretics, ACE inhibitors, and calcium channel blockers have been recommended [29]. Calcium channel blockers have also been found to be the most common antihypertensives used for management of hypertensive in this population and have been found to have good control rates [30]. Calcium channel is now recommended as a first line antihypertensive in the recent NICE guidelines [31]. Hence, management with oral antihypertensives is in accordance with the recommendations.

The reduction in systolic and diastolic blood pressure reported in our study shows a smooth decline in order to avoid risks of potential side effects of a much rapid decline. It is recommended that the initial reduction in mean arterial pressure (MAP) in case of hypertensive emergency should not be more than 20%–25% below the pretreatment blood pressure or that MAP be reduced within the first 30–60 minutes to 110–115 mm Hg [32]. This can be comparable to our study which reported an overall decrease of blood pressure of about 52 mm Hg in SBP from intravenous medication from the time of admission until 2 hours after admission in ER. A rapid decline in blood pressure is associated with acute deterioration in renal function, ischemic, cardiac, or cerebral events, and occasional retinal artery occlusion and acute blindness. The goal is to reduce BP by 10–15% over a period of 30–60 minutes, with the exception of the patient that presents with aortic dissection or acute intracranial bleed [33]. However treatment should be individualized to each patient based on the type and extent of end-organ damage, degree of BP elevation, and the specific side effects that each medication could have on a patient's preexisting comorbidities [34].

The strength of this study is that it is the first study from this region to report figures on prevalence of hypertensive crisis, as well as prevalence of complications as a result of hypertensive crisis among patients. Moreover, this study also reports management of these patients in terms of the treatment received in the ER and into the ward and their mean length of stay. However there are limitations in this study; firstly it has limited external validity as the sample is not representative for an entire population. It represents population visiting a single-tertiary care hospital and, hence, it is not representative of the entire population of Pakistan. Secondly, compliance issues with the medication regimen have not been taken into consideration; hence, the medications administered to the patients may be misjudged. Thirdly, some patients may also have gotten discharged against medical advice; hence, the optimal length of stay for these patients may not have been achieved. Fourth, this retrospective study cannot strongly give a cause and effect association and we have not reported the hypertensive urgencies and emergencies separately. We did not report data separately on hypertensive emergency and urgency and retinopathy could not be examined and reported in all patients.

## 5. Conclusion

The prevalence of hypertensive crisis is high in our subjects. Per oral calcium channel blocker and intravenous nitrate are the most commonly administered medications in the ER and ward. Acute renal failure is the most common complication developed in hypertensive crisis.

## Figures and Tables

**Table 1:** Baseline characteristics of patients overall, with hypertensive crisis and without hypertensive crisis (*N* = 387).

Characteristics	Overall	Hypertensive crisis	No hypertensive crisis	*P* value∗
	*N* = 387	*N* = 218 (56.3)	*N* = 169 (43.7)	
	*N* (%)	*N* (%)	*N* (%)	
Mean age (SD)	56.72 (14.78)	55.92 (15.09)	57.75 (14.36)	0.22
Male gender	175 (45.2)	89 (41)	86 (50.6)	0.03
Smoking	54 (14.0)	34 (15.6)	20 (11.8)	0.18
Dyslipidemia	167 (43.2)	87 (39.9)	80 (47.3)	0.08
Diabetes mellitus	143 (37.0)	77 (35.3)	66 (39.1)	0.25
Ischemic heart disease	83 (21.4)	34 (15.6)	49 (29)	0.001
Cerebrovascular accident	26 (6.7)	14 (6.4)	12 (7.1)	0.47

^*^ *P* value < 0.05 was taken as significant; it is calculated for hypertensive crisis and no hypertensive crisis.

**Table 2:** Comparison of blood pressure in patients with and without hypertensive crisis on intravenous and per oral medications.

	Hypertensive crisis			No hypertensive crisis		
	*N* = 218			*N* = 169		
	PO	IV		PO	IV	
	Mean (SD)	Mean (SD)	*P* value	Mean (SD)	Mean (SD)	*P* value
	*N* = 86	*N* = 130		*N* = 118	*N* = 51	
SBP 0∗	195 (17)	207 (17)	<0.001	156 (14)	162 (10)	0.01
SBP discharge∗∗	151 (27)	154 (23)	0.56	141 (19)	144 (19)	0.31
Drop in SBP∗∗∗	43 (27)	53.1 (29)	0.01	15 (21)	17.6 (21)	0.49
DBP 0	103 (15)	111 (17)	0.001	87 (15)	88 (14)	0.62
DBP discharge	85 (19)	85 (16)	0.93	77 (11)	79 (14)	0.46
Drop in DBP	17.8 (22)	25.8 (19)	0.006	9.6 (a6)	9.3 (15)	0.90

∗SBP 0 is systolic blood pressure on admission to ER, ∗∗SBP discharge was systolic blood pressure at discharge from ER or shifting to ward, and ∗∗∗drop in SBP was SBP on admission to ER-SBP at discharge from ER.

**Table 3:** Mean drop in systolic and diastolic blood pressure in ER in patients with hypertensive crisis according to type of intravenous medication.

Intravenous medication	Mean (SD) drop in SBP∗	*P* value∗∗	Mean (SD) drop in DBP	*P* value
	*N* = 218			
No IV medication	43.16 (27.9)	0.03	17.54 (22.42)	0.009
Nitrate	58.86 (29.5)		29.86 (22.48)	
Sodium nitroprusside	80 (15.5)		37.50 (7.77)	
Labetalol	50.62 (29.2)		27.11 (17.42)	
Hydralazine	48.18 (30.1)		18.50 (16.15)	
Beta blocker	45.21 (25.7)		18.71 (17.95)	

∗Drop in SBP was SBP on admission to ER-SBP at discharge from ER.

∗∗ *P* value is for comparison among the different intravenous medications.
